# CRISPR-based Activation of Endogenous Neurotrophic Genes in Adipose Stem Cell Sheets to Stimulate Peripheral Nerve Regeneration

**DOI:** 10.7150/thno.36790

**Published:** 2019-08-14

**Authors:** Mu-Nung Hsu, Han-Tsung Liao, Vu Anh Truong, Kai-Lun Huang, Fu-Jen Yu, Hwei-Hsien Chen, Thi Kieu Nuong Nguyen, Pavel Makarevich, Yelena Parfyonova, Yu-Chen Hu

**Affiliations:** 1 Department of Chemical Engineering, National Tsing Hua University, Hsinchu, Taiwan, 300; 2 Department of Plastic and Reconstructive Surgery, Craniofacial Research Center, Chang Gung Memorial Hospital, Linkou, Taiwan, 333; 3 College of Medicine, Chang Gung University, Taoyuan, Taiwan, 333; 4 Department of Plastic surgery, Xiamen Chang Gung hospital, China 361028; 5 Center for Neuropsychiatric Research, National Health Research Institutes, Miaoli, Taiwan, 350; 6 Laboratory of Gene and Cell Therapy, Institute for Regenerative Medicine, Lomonosov Moscow State University, Moscow, Russia 119192; 7 Laboratory of Angiogenesis, National Medical Research Center for Cardiology, Moscow, Russia 121152; 8 Laboratory of Postgenomic Technologies in Medicine, Faculty of Medicine, Lomonosov Moscow State University, Moscow, Russia 119192; 9 Frontier Research Center on Fundamental and Applied Sciences of Matters, National Tsing Hua University, Hsinchu, Taiwan 300

**Keywords:** CRISPR activation, adipose-derived stem cell, baculovirus, cell sheet, neurotrophic factor, nerve regeneration

## Abstract

**Background**: Peripheral nerve regeneration requires coordinated functions of neurotrophic factors and neuronal cells. CRISPR activation (CRISPRa) is a powerful tool that exploits inactive Cas9 (dCas9), single guide RNA (sgRNA) and transcription activator for gene activation, but has yet to be harnessed for tissue regeneration.

**Methods**: We developed a hybrid baculovirus (BV) vector to harbor and deliver the CRISPRa system for multiplexed activation of 3 neurotrophic factor genes (*BDNF*, *GDNF* and *NGF*). The hybrid BV was used to transduce rat adipose-derived stem cells (ASC) and functionalize the ASC sheets. We further implanted the ASC sheets into sciatic nerve injury sites in rats.

**Results**: Transduction of rat ASC with the hybrid BV vector enabled robust, simultaneous and prolonged activation of the 3 neurotrophic factors for at least 21 days. The CRISPRa-engineered ASC sheets were able to promote Schwann cell (SC) migration, neuron proliferation and neurite outgrowth *in vitro*. The CRISPRa-engineered ASC sheets further enhanced *in vivo* functional recovery, nerve reinnervation, axon regeneration and remyelination.

**Conclusion**: These data collectively implicated the potentials of the hybrid BV-delivered CRISPRa system for multiplexed activation of endogenous neurotrophic factor genes in ASC sheets to promote peripheral nerve regeneration.

## Introduction

Peripheral nerve injuries can lead to disability and remain a challenge in the clinical setting 1. Traumatic injury to peripheral nerves elicits disconnection of distal and proximal stumps, macrophage/monocyte infiltration to remove myelin and axon debris, and subsequent neuronal degeneration 2. Yet the injury also stimulates proliferation of Schwann cells (SC), which are the major support cells mediating axon remyelination and peripheral nerve regeneration. Although peripheral nerve injuries can be treated by end-to-end suturing or autologous nerve grafting 3, peripheral nerve regeneration is usually far from complete. Only less than 50% of patients regain full function after treatment 2.

Neurotrophic factors are a family of proteins that regulate the growth, survival, and differentiation of neurons 4. For instance, nerve growth factor (NGF) mediates survival and maturation of developing neurons in the peripheral nervous system 5. Brain-derived neurotrophic factor (BDNF) exerts neuroprotective and growth-promoting effects on neurons after injury and supports motor neuron survival in lesioned animals 5, 6. Glial cell line-derived neurotrophic factor (GDNF) triggers proliferation and migration of SC, promotes motor neuron survival and improves nerve regeneration 7. These 3 neurotrophic factors have been assessed for nerve regeneration and may be delivered into the nerve injury site as recombinant protein 3, 7 or by genetically engineered cells that overexpress the factor 8, 9.

Adipose-derived stem cells (ASC) are a promising cell-based gene delivery vehicle and can form cell sheets that consist of cells, cell-to-cell junctions and extracellular matrix 10 to promote peripheral nerve repair 11. ASC can also be genetically engineered by baculovirus (BV), which is an insect virus but can be adapted to deliver genes into ASC at efficiencies exceeding 95% 12, 13. We previously developed a hybrid BV system comprising two vectors: one expressing Cre recombinase (Bac-Cre) and the other substrate BV harboring the transgene cassette flanked by loxP sites 14. Co-transduction of ASC with the hybrid Cre/loxP-based BV system results in Cre-mediated excision of the transgene cassette off the substrate BV genome and formation of episomal DNA minicircle encompassing the transgene within the cells 14, 15. This hybrid BV system was shown to prolong and enhance transgene expression, and was exploited to express microRNA sponge or growth factor to augment ASC differentiation and bone healing *in vivo* 16, 17. Furthermore, the hybrid BV was used to engineer ASC sheet for prolonged GDNF expression, which after implantation into the sciatic nerve injury site in rats ameliorated the functional and histological recovery 18.

CRISPR-Cas9 is an RNA-guided gene editing system in which the ectopically expressed Cas9 nuclease and single guide RNA (sgRNA) orchestrate to recognize the target DNA by the programmable spacer sequence on the sgRNA and trigger DNA cleavage by Cas9 19, 20. CRISPR-Cas9 system was repurposed for CRISPR activation (CRISPRa) by using a catalytically inactive Cas9 (dCas9), which is fused with transcription activators such as VP64 and coordinate with the programmable sgRNA to stimulate target gene expression 21. The magnitude of stimulation was further enhanced using a synergistic activation mediator (SAM) system which consists of (i) dCas9 fused with VP64 (dCas9-VP64), (ii) chimeric sgRNA with two additional copies of MS2 RNA aptamers that bind with MS2 coat protein (MCP), and (iii) MPH fusion protein comprising MCP, p65 and heat shock factor 1 (HSF1) that recruit transcription factors as the activation complex 22. By designing multiple sgRNA, dCas9-VP64, sgRNA and MPH co-expressed in the same cell can associate together to activate up to 10 endogenous genes 22.

It has been shown that combined use of neurotrophic factors can synergistically improve functional recovery of nerve injury (for review see 6). Given the potentials of CRISPRa system for multiplexed gene activation and BV for ASC sheet engineering, here we aimed to develop a hybrid BV carrying the SAM-based CRISPRa system for simultaneous activation of 3 different neurotrophic factors in ASC sheets and nerve regeneration. The hybrid BV accommodated dCas9-VP64, MPH and 3 sets of sgRNA (3 sgRNA for each gene, 9 sgRNA in all) to target *BDNF*, *GDNF* and *NGF*. We showed that co-transduction of rat ASC with Bac-Cre and the hybrid BV robustly activated the expression of BDNF, GDNF and NGF. We further fabricated and functionalized the rat ASC sheet with the hybrid BV system. The functionalized ASC sheets co-expressing BDNF, GDNF and NGF improved SC migration, neuron regeneration and neurite outgrowth *in vitro*. Implantation of the functionalized ASC sheets into sciatic nerve injury sites further enhanced *in vivo* functional recovery, nerve reinnervation, axon regeneration and remyelination. These data collectively demonstrated that CRISPRa system delivered by the hybrid Cre/loxP-based BV enable multiplexed activation of endogenous neurotrophic genes in ASC sheet to promote peripheral nerve regeneration.

## 1. Materials and methods

### 1.1. Recombinant BV preparation

The recombinant BV (Bac-Cre) expressing the Cre recombinase was constructed previously 14. To construct the substrate BV Bac-LECW, we used pBacLEW 17 that contained two loxP sites as the backbone. Briefly, the *Streptococcus pyogenes dCas9* gene containing a 5' NLS (nuclear localization signal) was PCR-amplified from pcDNA-dCas9-p300 Core (Addgene #61357 23), by which an additional NLS was also appended to the 3' end. The *dCas9* gene with 5' and 3' flanking NLS was subcloned into pBacLEW downstream of the rat EF-1α promoter (with CMV enhancer) to generate pBac-dCas9. The activation domain of *VP64* gene was PCR-amplified from pHAGE EF1α dCas9-VP64 (Addgene #50918 24) and subcloned into pBac-dCas9, downstream of the *dCas9* sequence, to generate pBac-dCas9_VP64. In parallel, the *MCP-p65-HSF1* (MPH) fusion gene was PCR-amplified from pMS2-P65-HSF1_GFP (Addgene #61423 22) with a porcine teschovirus-1 2A (P2A) sequence at the 5' end. The MPH fusion gene was subcloned into pBac-dCas9_VP64 downstream of VP64 to yield pBac-dCas9_VP64_MPH.

To construct the sgRNA array, we PCR-amplified the sgRNA 2.0 cassette from sgRNA (MS2) cloning backbone (Addgene #61424 22) which consists of a human U6 (hU6) promoter, a spacer insertion linker, and the sgRNA scaffold with two MCP recognition aptamer sequences. The sgRNA cassette was subcloned into TA vector to yield pTA-sgRNA. The spacer sequences were designed using online tool (http://crispr-era.stanford.edu/) with the highest computing targeting efficiency and specificity score (*BDNF*: -75, -138 and -208; *GDNF*: -113, -225 and -466; *NGF*: -155, -186 and -691 from transcription start sites). The spacers were synthesized and cloned into pTA-sgRNA with *BbsI*, downstream of the hU6 promoter. After separate construction, 9 different sgRNA cassettes were assembled using comparable cohesive restriction sites (*Xba, NheI* and* XhoI*) to generate a sgRNA array and inserted into pBac-dCas9_VP64_MPH. The entire cassette (sgRNA array, dCas9-VP64, MPH) was flanked by two loxP sites. The resultant plasmid (pBac-LECW) was used to generate the recombinant BV (Bac-LECW) using Bac-to-Bac^®^ System (ThermoFisher Scientific). The BV stocks were produced by infecting insect cell Sf-9 cultured using TNM-FH medium (Sigma) and titered by end-point dilution method 15.

### 1.2. ASC isolation, ASC sheets preparation and transduction

All animal experiments were performed in compliance with the Guide for the Care and Use of Laboratory Animals (Ministry of Science and Technology, Taiwan). The experimental protocols were approved by the Institutional Animal Care and Use Committee of National Tsing Hua University. ASC were isolated from fat pads of 4-week old male Sprague-Dawley (SD) rats (BioLasco, Taiwan) as described in Supporting Info. ASC were passaged 3 to 5 times for following experiments.

The ASC sheets fabrication and transduction were performed as described 18 with minor modification. Rat ASC were seeded to 6-well plates (2×10^6^ cells/well) and cultured at 37ºC with α-MEM containing 20% fetal bovine serum (FBS), 100 IU/ml penicillin and 100 IU/ml streptomycin. After 24 h, ASC sheets were formed and gently washed with phosphate-buffered saline (PBS). The BV solution was diluted with fresh TNM-FH medium depending on the multiplicity of infection (MOI). The virus solution was mixed with transduction medium (NaHCO_3_-free α-MEM) at a volumetric ratio of 1:4 and added to the wells (500 μl/well). For mock-transduction control, fresh TNM-FH medium was mixed with virus-free, NaHCO_3_-free α-MEM at a volumetric ratio of 1:4 and added to the wells. The plates were gently shaken on a rocking plate for 6 h, and the transduction mixture was removed, followed by culture at 37ºC with α-MEM medium containing 3 mM sodium butyrate and 20% FBS. At 48 h post-seeding, the transduced ASC sheets were washed with PBS twice and briefly trypsinized for 10 sec using 0.05% trypsin-EDTA (0.5 ml/well, Gibco). After trypsin removal, ASC sheets were washed twice, incubated in PBS and gently shaken. The transparent ASC sheets spontaneously detached from the well and were handled with a forceps for subsequent experiments.

### 1.3. Measurement of neurotrophic factors expression levels

Rat ASC were seeded to 6-well plates (5×10^5^ cells/well) and cultured overnight with α-MEM medium containing 10% FBS. The cells were co-transduced with Bac-Cre/Bac-LECW (MOI 100/150) or mock-transduced as described above. After 6 h, the transduction solution was removed and ASC were cultured with α-MEM medium containing 3 mM sodium butyrate and 10% FBS. After 18 h, the medium was replaced with fresh α-MEM containing 10% FBS and continued to be cultured. Half of the medium was exchanged every 48 h with fresh α-MEM medium containing 10% FBS. The neurotrophic factor concentrations in the culture supernatant were analyzed using ELISA kits (Raybiotech® ELR-BDNF-1, ELR-GDNF-1 or ELR-bNGF-1).

### 1.4. SC migration assay

The ability of ASC sheets to recruit SC was verified by the transwell co-culture assay. Rat ASC sheets were mock-transduced or co-transduced with Bac-Cre/Bac-LECW as above and detached. One sheet (≈2×10^6^ cells/sheet) was transferred and cultured at the bottom of the 24-well transwell plates (Corning) while rat SC (Bioresource Collection and Research Center, Taiwan) were seeded to the top porous inserts (pore diameter=0.4 μm) at 5×10^5^ cells/well. The rat ASC and SC were co-cultured with α-MEM medium containing 10% FBS, 100 IU/ml penicillin and 100 IU/ml streptomycin. Two days after co-culture, the porous inserts were washed, stained with crystal violet and photographed. The numbers of migrating SC were counted using ImageJ software (NIH, USA).

### 1.5. Scratch assay

Rat dorsal root ganglion (DRG) neurons were isolated as described in Supporting Info, seeded onto cover glasses (5×10^5^ cells/glass) and cultured using DMEM (containing 10% FBS, 100 IU/ml penicillin, 100 IU/ml streptomycin and 10 ng/ml NGF) in the 6-well plate. After two days, the cover glasses were transferred to a new 6-well plate and co-cultured with ASC sheets that were mock-transduced or co-transduced with Bac-Cre/Bac-LECW (MOI 100/150). Five days after co-culture, DRG neurons on the cover glass were washed with PBS and subjected to immunolabeling. The primary antibody was specific for neuron marker NeuN (1:500 dilution, Abcam) while the secondary antibody was conjugated with Alexa Fluor^®^ 488 (1:200 dilution, Abcam). After staining, the neuron proliferation/extension were observed under the fluorescence microscope and the neuron number/average neurite length were analyzed using ImageJ software.

### 1.6. Implantation of ASC sheets into sciatic nerve injury sites

Twenty-eight female Sprague-Dawley rats (8-weeks old, BioLasco, Taiwan) were randomly divided into 4 groups and anesthetized by intramuscular injection of Zoetil^TM^ (10 mg/kg body weight, Virbac) and Rompun^TM^ (0.2 mg/kg body weight, Bayer). Sham group (*n*=4) rats underwent no surgery while all other rats underwent surgery. After skin incision, the right hind limb sciatic nerve was exposed and transected with microsurgery scissors. The proximal and distal ends were directly sutured with the 10-0 VICRYL^TM^ suture (Ethicon), and the injury site were directly wrapped with 2 ASC sheets that were mock-transduced (Mock group, *n*=8) or co-transduced with Bac-Cre/Bac-LECW at MOI 100/150 (CRISPRa group, *n*=8). As a negative control, the injury site received no ASC sheets (NC group, *n*=8). The limb was closed with a 4-0 Polysorb^TM^ suture and injected with 300 μl saline for fluid supplement and 250 μl gentamycin (1 mg/ml). Neomycin ointment was also administered.

### 1.7. Walking gait analysis

At 4, 6 and 8 weeks after surgery, we performed walking gait analysis using a high speed video-assisted analysis system (GaitScan System, CleverSys Inc) as described 18. The rats walked across an acrylic tunnel and the walking gaits were recorded by the monitor system at the bottom of the tunnel. Both step length (the distance between each step to the contralateral hind limb) and stride length (the distance between two successive placements of the same foot) were measured. Each parameter was averaged from at least 10 footsteps.

### 1.8. Electrophysiological evaluation by electromyography (EMG)

At 8 weeks post-surgery, rats were anesthetized using Zoetil^TM^ and the sciatic nerves were carefully exposed. The monopolar needle electrode connected to a DC electrical stimulator was placed on the proximal end of the nerve, while the collector needle electrode was placed 15 mm away from the stimulator needle electrode in the gastrocnemius muscle. The nerve was stimulated (0.5-2.0 Hz) and the compound muscle action potential of the gastrocnemius muscle was recorded (MP35, Biopac System). The peak-to-peak amplitude (p-p), maximum peak amplitude (p max) and time to deflection (latency) were determined from the electromyogram using the Student Lab PRO software (Biopac).

### 1.9. Wet muscle weight measurement and histological examination

The rats were further euthanatized by CO_2_ overdose after EMG analysis and gastrocnemius muscles in the left and right hind limbs were dissected, detached from the bone and weighed immediately. In parallel, the sciatic nerves were harvested, fixed in 4% phosphate-buffered paraformaldehyde, embedded in paraffin (Bio-Check Laboratories) and sectioned (5 μm). The sections in the distal end of the regenerated nerve (≈30 μm distal to the injury site) were subjected to immunofluorescence staining. The antigen in the sections were retrieved by incubating with 0.1% trypsin at 37ºC for 30 min and cooled down at room temperature for 10 min, followed by 2 PBS washes. The sections were blocked with 1% bovine serum albumin (BSA) in PBS at room temperature for 2 h, washed with PBS twice and were incubated with primary antibody against S100 (1:200 dilution, Abcam) or neurofilament (1:200 dilution, Abcam) for 1 h. After washing, the sections were incubated with Alexa Fluor^®^ 488-conjugated secondary antibody (1:500 dilution, Abcam) at room temperature for 1 h. Following 2 PBS washes, the presence of S100 or neurofilament was photographed using the fluorescence microscope. The regenerated axon (neurofilament, pixel^2^) area and remyelination (S100, pixel^2^) area were analyzed using ImageJ.

### 1.10 Statistical analysis

All *in vitro* data were representative of at least 3 independent culture experiments. All quantitative data were analyzed using student's *t*-test and are expressed as means±standard deviations (SD). *p*< 0.05 was considered significant.

## 2. Results

### 2.1. Design and construction of CRISPRa system

**Fig 1:**
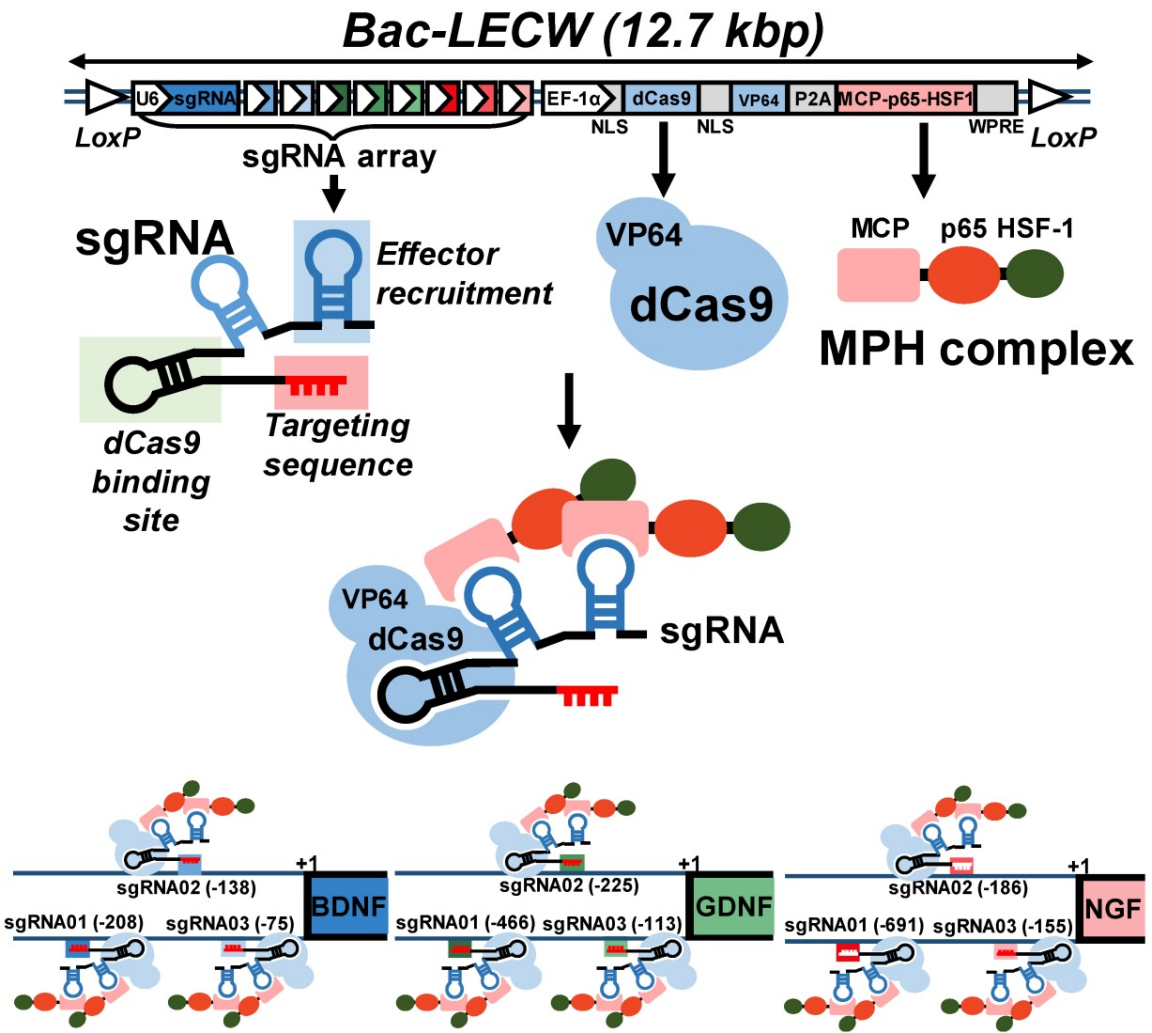
Illustration of hybrid BV vector (Bac-LECW) carrying the CRISPRa system for neurotrophic factor gene activation. Bac-LECW harbored *dCas9* (with 5' and 3' NLS sequences) fused with *VP64* activation domain (dCas9-VP64) and the MPH (MCP-p65-HSF1) activation effector. *dCas9*-*VP64* and MPH were linked with P2A sequence and co-expressed under the control of rat EF-1α promoter. Bac-LECW also harbored 9 sgRNA expression cassettes each consisting of the hU6 promoter, sgRNA 2.0 backbone with two MCP binding motifs and the spacer sequences targeting different sequences on the neurotrophic factor genes (*BDNF*: -75, -138 and -208; *GDNF*: -113, -225 and -466; *NGF*: -155, -186 and -691). The entire cassettes were flanked by two loxP sites. Co-transduction of ASC with Bac-Cre and Bac-LECW would lead to prolonged expression of dCas9-VP64 and 9 different sgRNA, which coordinate to target different positions of *BDNF*, *GDNF* and *NGF* genes and recruit the MPH effector to activate gene expression.

To explore the CRISPRa system for simultaneous activation of multiple neurotrophic genes, we chose the SAM system 22 and first constructed a cassette expressing a dCas9-VP64 fusion protein and an MPH activation effector consisting of MCP, p65 and HSF1 (Fig. 1). We next selected *BDNF*, *GDNF* and *NGF* as the target genes and constructed a sgRNA array comprising 9 sgRNA expression cassettes, with each gene targeted by 3 sgRNA (Fig. 1). Each sgRNA consisted of two MS2 aptamer motifs for MPH effector recruitment and targeting sequences to bind the target genes. To facilitate the co-delivery of dCas9-VP64, MPH and sgRNA into the same cells, we combined the sgRNA array cassettes with the cassette expressing dCas9-VP64 and MPH, and packaged the entire cassette (≈12.7 kb) into BV vector to yield a recombinant BV Bac-LECW (Fig. 1). To exploit the Cre/loxP-based hybrid BV system, the vector contained two loxP sites to flank the entire cassette. We envisioned that co-transduction of ASC with Bac-Cre (expressing Cre recombinase) and Bac-LECW would lead to Cre-mediated minicircle formation and prolong the expression of dCas9-VP64 and 9 different sgRNA, which coordinate to target different positions of *BDNF*, *GDNF* and *NGF* genes and recruit the MPH effector to activate gene expression (Fig. 1).

### 2.2. BV-expressed CRISPRa system activated neurotrophic gene expression

**Fig 2:**
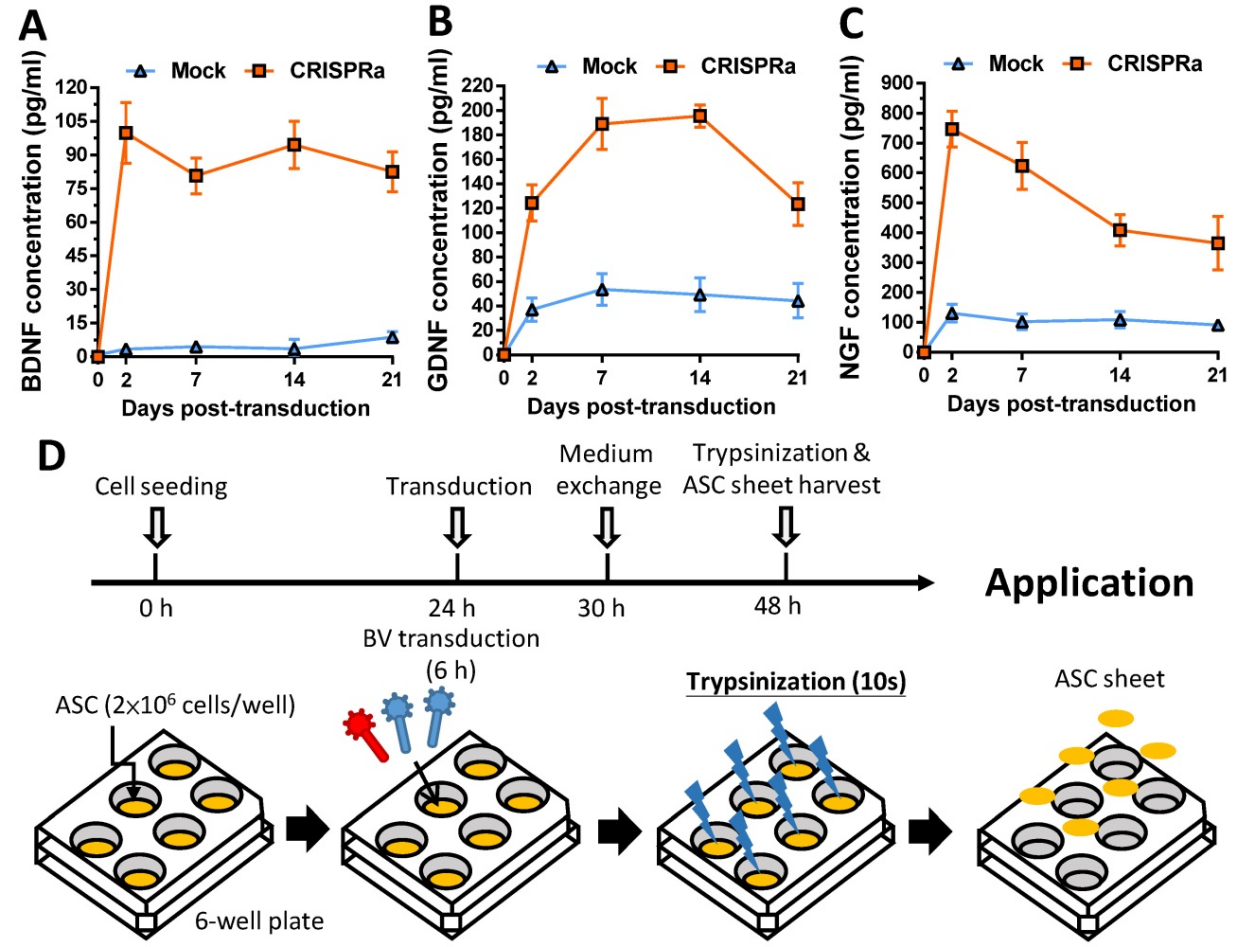
Confirmation of prolonged gene activation in ASC transduced by the hybrid BV and preparation of BV-transduced ASC sheets. (A-C) Expression profile of BDNF (A), GDNF (B) and NGF (C). (D) Fabrication and transduction of rat ASC sheets. The data represent means±SD of 3 independent culture experiments.

After BV construction, we mock-transduced (Mock group) or co-transduced rat ASC with Bac-Cre and Bac-LECW (CRISPRa group) and analyzed the expression of BDNF, GDNF and NGF by ELISA. Figs. 2A-2C confirm that CRISPRa group remarkably activated the expression of all 3 neurotrophic factors at 2 days post-transduction (dpt) as opposed to the Mock control, reaching 99.9±11.7 pg/ml (31.3±8.6 Fold) for BDNF, 124.2±12.8 pg/ml (3.6±1.2-fold) for GDNF and 746.8±52.2 pg/ml (5.9±0.8-fold) for NGF. Even at 21 dpt, the CRISPRa group expressed significantly more BDNF (9.9±2.2-fold, 82.6±7.7 pg/ml), GDNF (3.2±1.7-fold, 123.4±15.1 pg/ml) and NGF (4.1±1.1-fold, 365.0±77.6 pg/ml) than the Mock group. These data confirmed that the BV-delivered CRISPRa system activated the expression of all 3 target genes in ASC for a prolonged period of time.

To apply CRISPRa-engineered ASC for nerve regeneration, we fabricated and functionalized the rat ASC sheets by BV transduction as described 18 with minor modifications (Fig. 2D). We seeded rat ASC (2×10^6^ cells/well) onto 6-well plates at 0 h, cultured the cells using 20% FBS and co-transduced the ASC sheets with Bac-Cre and Bac-LECW 24 h later. As a control, the ASC sheets were also mock transduced (Mock group). After 6 h of transduction, we performed medium exchange and harvested the ASC sheets at 48 h by brief trypsinization (Fig. 2D).

### 2.3. CRISPRa-stimulated neurotrophic gene expression triggered SC recruitment, neuron proliferation and neurite growth

**Fig 3:**
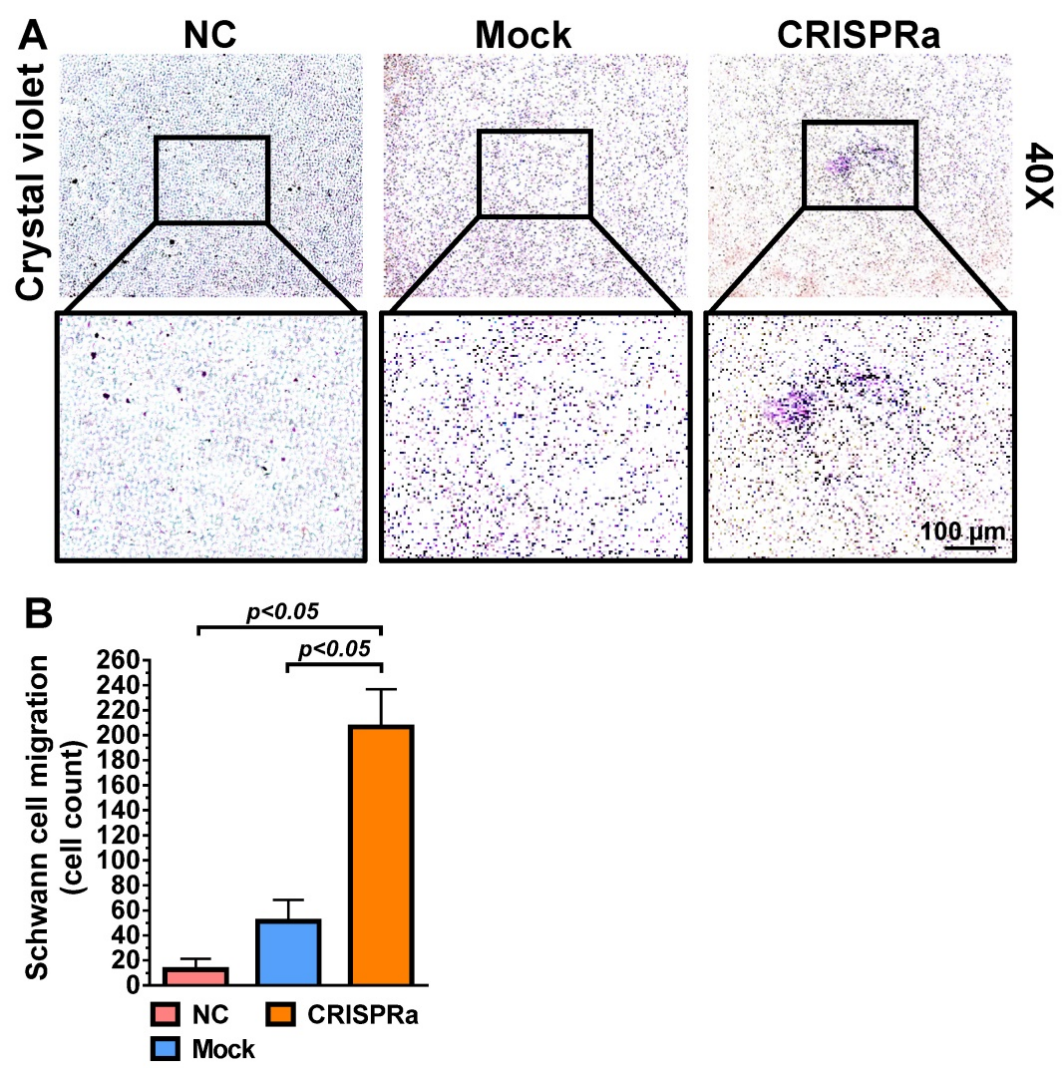
CRISPRa-engineered ASC sheets promoted SC migration. (A) SC migration induced by the CRISPRa-engineered ASC sheets. (B) Number of SC migrating through the membrane. Rat ASC sheets cultured in 6-well plates were co-transduced with Bac-Cre and Bac-LECW at MOI 100/150 (CRISPRa group) or mock-transduced (Mock group) and then co-cultured with rat SC in the transwell assay. Two days after co-culture, the porous inserts were stained with crystal violet. Four fields in each insert were randomly chosen and the numbers of migrating SC were counted using ImageJ software. The data represent means±SD of 3 independent culture experiments.

Since SC are important support cells for nerve regeneration, whether the transduced ASC sheets were functionalized was first verified by a transwell co-culture assay. The mock-transduced (Mock group) and Bac-Cre/Bac-LECW-transduced (CRISPRa group) rat ASC sheets were placed at the bottom of well and co-cultured with rat SC at the top membrane filter. In parallel, rat SC were cultured alone at the membrane filter as a negative control (NC group). Two days later, crystal violet staining illustrated no obvious SC migration through the membrane in the NC group and only slight SC migration in the Mock group. In contrast, the CRISPRa group induced perceptible SC migration (Fig. 3A). Quantitative analysis showed that the CRISPRa group triggered more evident (≈15.0±2.9-fold) SC migration than the NC group (Fig. 3B), indicating that Bac-Cre/Bac-LECW-transduced ASC sheets secreted functional neurotrophic factors capable of recruiting SC.

**Fig 4:**
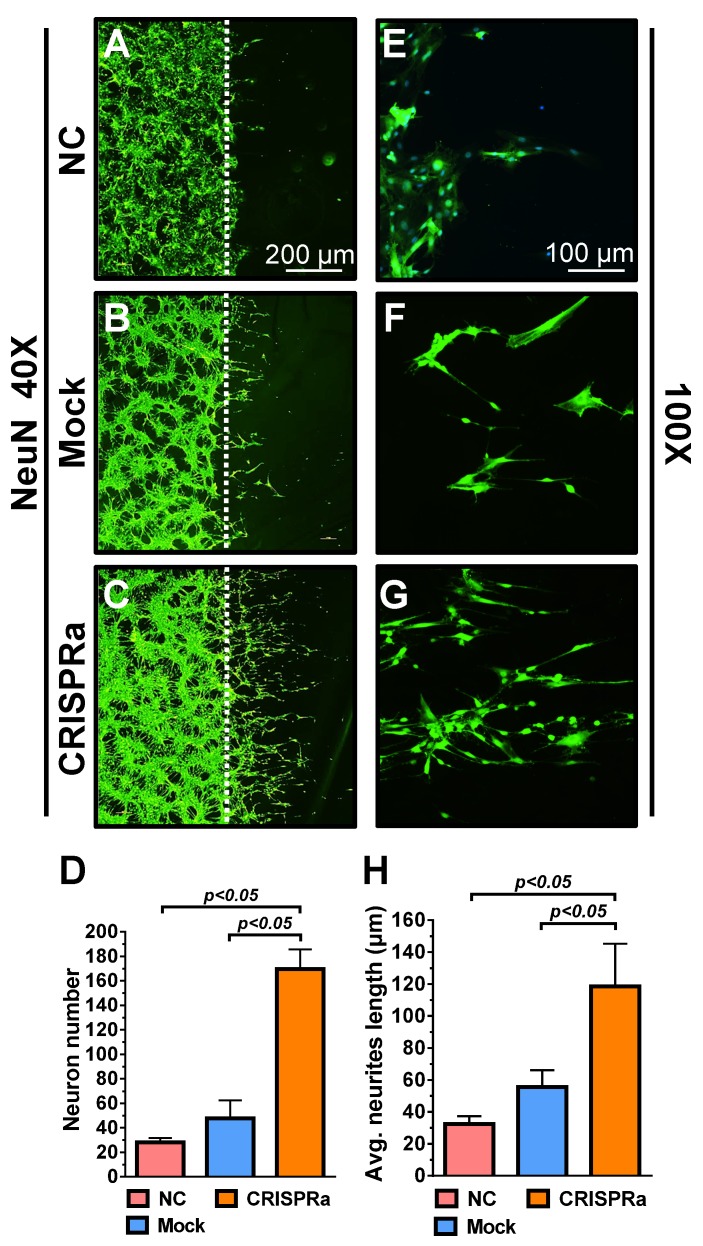
CRISPRa-engineered ASC sheets stimulated neuron proliferation and neurite outgrowth. (A-F) DRG neuron proliferation and extension as detected by immunolabeling specific for NeuN. (G) Neuron numbers. (H) Average neurite length. DRG neurons were seeded onto cover glasses (5×10^5^ cells/glass) for 2 days and a wound gap was created using a cell scrapper. The neurons on the cover glass were transferred to a new 6-well plate and cultured alone as a negative control (NC group) or co-cultured with the mock-transduced (Mock group) or Bac-Cre/Bac-LECW-transduced (MOI=100/150, CRISPRa group) rat ASC sheets. After 5 days, the neurons were subjected to NeuN-specific immunostaining. Four fields in each cover glass were randomly chosen and the numbers of neurons extending through the gap were counted using ImageJ software. The average neurite lengths were also calculated using ImageJ software. The data represent means±SD of 3 independent culture experiments.

Whether the neurotrophic factors could stimulate neuron proliferation and extension was examined by a neuron scratch assay. Dorsal root ganglion (DRG) neurons were seeded on the cover glass for 2 days and a wound gap was created using a cell scrapper. The neurons on the cover glass were transferred to a new 6-well plate and cultured alone as a negative control (NC group) or co-cultured with the mock-transduced (Mock group) or Bac-Cre/Bac-LECW-transduced (CRISPRa group) rat ASC sheets. Five days later, neuron proliferation through the wound gap was barely observed in the NC group and was marginal in the Mock group, but was apparent in the CRISPRa group, as judged from the immunolabeling specific for neuron marker NeuN (Figs. 4A-4C). Quantitative analysis showed that, compared with the NC group, the CRISPRa group elicited 6.1±0.5-fold neuron proliferation (Fig. 4D). The enlarged views of the neurites (Figs. 4E-4G) and quantitative analysis (Fig. 4H) of average neurite length depicted significantly (*p*<0.05) more evident neurite outgrowth in the CRISPRa group than in the Mock and NC groups. These data attested that CRISPRa-induced neurotrophic factors expression stimulated neuron proliferation and neurite outgrowth.

### 2.4. Implantation of CRISPRa-engineered ASC sheets enhanced in vivo functional recovery

**Fig 5:**
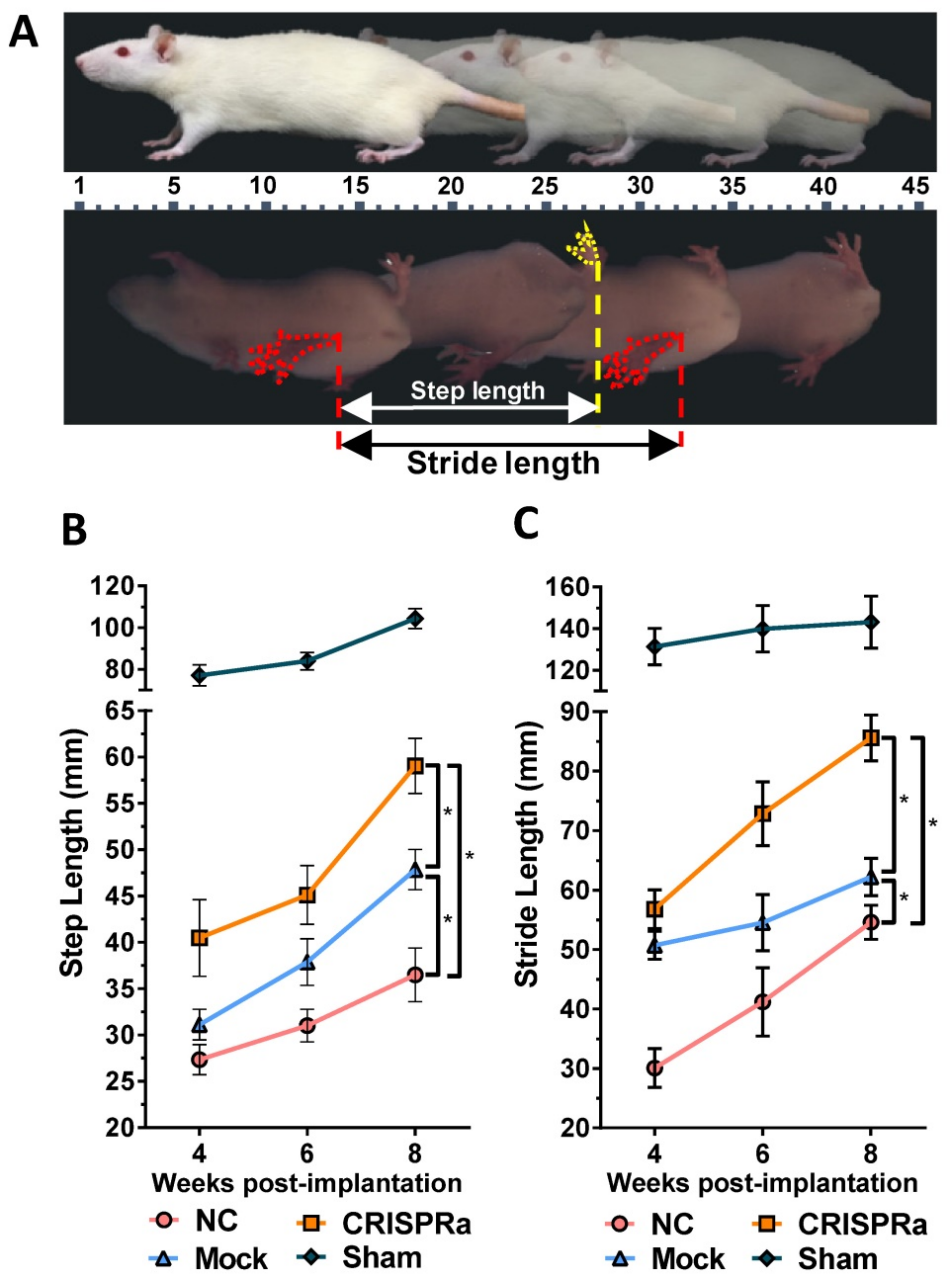
CRISPRa-engineered ASC sheets enhanced *in vivo* functional recovery. (A) Illustration of walking gait analysis and step/stride lengths. (B) Step length. (C) Stride length. Three groups of rats underwent sciatic nerve transection and implanted with (1) no ASC sheets (NC group, *n*=8); (2) mock-transduced ASC sheets (Mock group, *n*=8) or (3) ASC sheets co-transduced with Bac-Cre/Bac-LECW (MOI 100/150, *n*=8, CRISPRa group). The rats underwent no surgery served as the control (Sham group, *n*=4). The step length and stride length were measured and averaged from at least 10 footsteps. *p<0.005.

To evaluate the effectiveness of CRISPRa-engineered ASC sheets for nerve regeneration, three groups of rats underwent sciatic nerve transection at the right hind limb and were implanted with (1) no ASC sheet for direct repair (*n*=8, NC group); (2) mock-transduced ASC sheets (*n*=8, Mock group) or (3) ASC sheets co-transduced with Bac-Cre/Bac-LECW (*n*=8, CRISPRa group, Fig. S1). In parallel, another group of rats undergoing no surgery served as a positive control (*n*=4, Sham group). Walking gait analysis (Fig. 5A) revealed that the step length (61.1±7.5 mm) and stride length (85.6±13.2 mm) of the CRISPRa group at week 8 were 73.9±6.8% and 68.2±16.1% higher than those at week 4, respectively. Moreover, at week 8 the step length and stride length of the CRISPRa group significantly (*p*<0.05) exceeded those of the Mock and NC groups, attesting that the CRISPRa-engineered ASC sheets improved the functional recovery.

### 2.5. CRISPRa-engineered ASC sheets enhanced nerve reinnervation and prevented muscle atrophy

**Fig 6:**
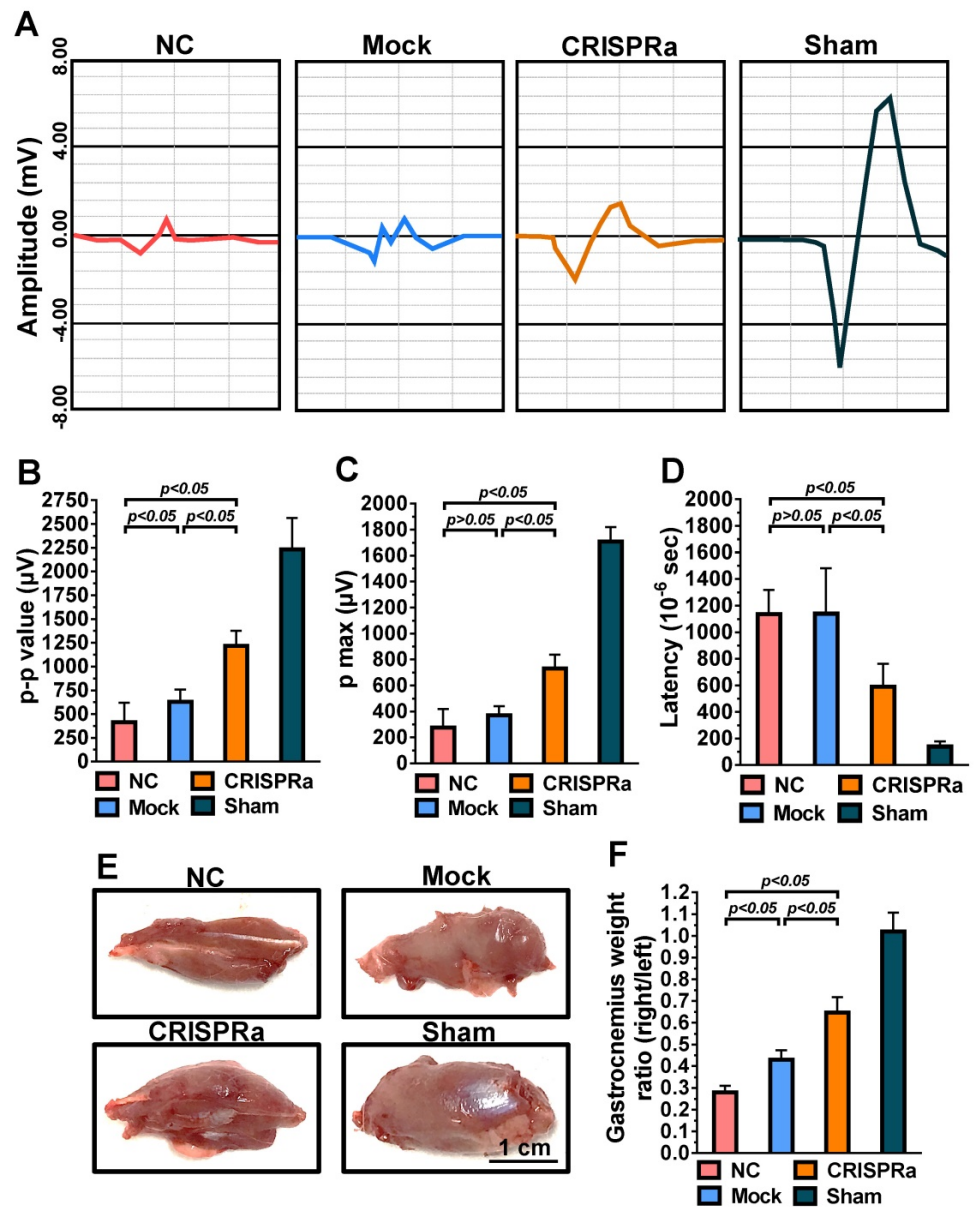
CRISPRa-engineered ASC sheets enhanced nerve reinnervation and prevented muscle atrophy. (A) CMAP recorded by EMG. (B) p-p amplitude. (C) p max amplitude. (D) Latency. (E) Representative photos of gastrocnemius muscles. (F) Wet weight ratios of gastrocnemius muscle (right vs. left hind limb). At 8 weeks post-implantation, the CMAP of the gastrocnemius muscle was recorded. The p-p amplitudes, p max amplitudes and latency values were determined. After EMG analysis, the gastrocnemius muscles were removed from the right and left hind limbs, weighed and compared.

Functional recovery after peripheral nerve injury is dependent on the amount and accuracy of nerve reinnervation 25. Since gastrocnemius is the largest muscle innervated by the sciatic nerve in rats, we further examined the sciatic nerve conduction by measuring the compound muscle action potentials (CMAP) of gastrocnemius muscle at week 8 by electromyography (EMG). As shown in Fig. 6A, the wave shape of CMAP was more intact in the CRISPRa group than in the NC and mock groups, suggesting the improvement of overall nerve regeneration. Since higher EMG amplitudes suggest more reinnervated motor neurons while lower latency indicates faster conduction velocity, we quantified the peak-to-peak amplitude (p-p), maximum peak amplitude (p max) and deflection time (latency) to evaluate the degree of reinnervation. The p-p (Fig. 6B), p max (Fig. 6C) and latency (Fig. 6D) values of the Mock and NC groups were statistically similar (*p*>0.05), suggesting that mock-transduced ASC sheets alone did not improve the nerve reinnervation. In contrast, the CRISPRa group conferred significantly (*p*<0.05) higher p-p and p-max as well as lower latency values (Figs. 6B-D) than the Mock and NC groups, indicating that the CRISPRa-engineered ASC sheets augmented reinnervation and electrophysiological functionality.

Damage of sciatic nerve results in loss of neural innervation to the gastrocnemius muscle and hence muscle atrophy 26. After EMG analysis, the rat gastrocnemius muscle was dissected for evaluation of muscle atrophy. The representative photos (Fig. 6E) illustrated obvious atrophy on the transected side in the NC and Mock groups, but not in the CRISPRa group. The degree of reinnervated muscle weight was assessed by the wet weight ratios of the gastrocnemius (right vs. left hind limb). Fig. 6F depicts that the CRISPRa group conferred significantly (*p*<0.05) higher gastrocnemius weight ratios than the NC and Mock groups, demonstrating the effectiveness of the CRISPRa-engineered ASC sheets in preventing muscle atrophy.

### 2.6. CRISPRa-engineered ASC sheets enhanced axon regeneration and remyelination

**Fig 7:**
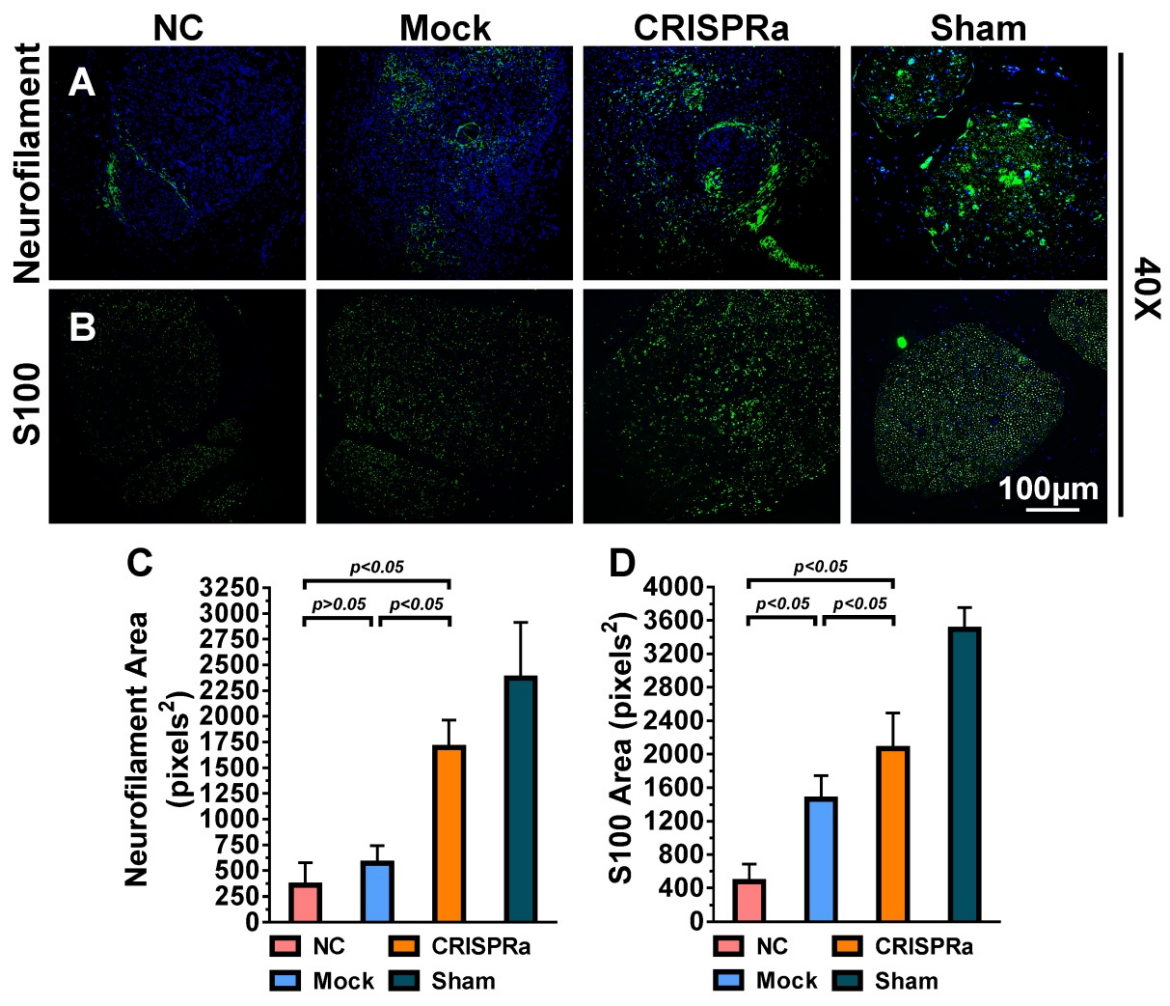
CRISPRa-engineered ASC sheets enhanced axon regeneration and remyelination. (A-B) Immunolabeling specific for axon marker neurofilament (A) and myelin-forming SC marker S100 (B). (C) Area of neurofilaments (pixels^2^). (D) Area of S100 (pixels^2^). For each group, 5 sections near the transection site were immunolabeled and 5 fields from each section were analyzed using the ImageJ software. Representative images are shown and the data represent means±SD.

Successful nerve regeneration hinges on axons regeneration and remyelination, thus we sectioned the distal nerve at week 8 and performed histopathology analysis. Immunolabeling specific for the axon marker neurofilament (Fig. 7A) illustrated that the axon regeneration was scarce in the NC and Mock groups but increased in the CRISPRa group. Immunolabeling specific for S100, a marker of myelin-forming SC, revealed that SC/myelin sheaths scarcely existed in the NC group but were more evident in the CRISPRa group (Fig. 7B). Quantitative image analysis confirmed that the CRISPRa-engineered ASC sheets endowed significantly (*p*<0.05) higher degrees of axon regeneration (Fig. 7C) and remyelination (Fig. 7D) than the NC and Mock groups. These data altogether confirmed that the expressed neurotrophic factors by the CRISPRa-functionalized ASC sheets stimulated axon regeneration and remyelination.

## 3. Discussion

CRISPRa technology has been exploited for programmable gene activation for genome-wide genetic screening 27, identification of long non-coding RNA involved in drug resistance 28 and *in vitro* cell reprogramming. With regard to cell fate manipulation *in vitro*, CRISPRa has been harnessed to convert mouse embryonic fibroblasts to neuronal cells 29, reprogram mouse embryonic 30 or human skin 31 fibroblasts to induced pluripotent stem cells (iPSC), accelerate the differentiation of human iPSC into neurons and astrocytes 32, as well as induce mesenchymal stem cell differentiation into adipocyte-like cells 33. Moreover, CRISPRa has been employed to treat muscular dystrophy symptoms in mouse models 34. However, CRISPRa has yet to be harnessed for nerve regeneration *in vivo*.

The earliest CRISPRa was developed by fusing dCas9 with the transcription activator VP64 21. Furthermore, dCas9 may be fused with a tandem array of peptides 35, epigenome modifier 23 or with a tripartite activator VPR 36 to enhance the magnitude of stimulation. Here we exploited the SAM system 22 to simultaneously active 3 neurotrophic genes in ASC sheets in order to promote nerve regeneration. We chose the SAM system because it was proven to be superior or at least comparable to other CRISPRa systems with regard to activation magnitude 37, and SAM system was already used to treat muscular dystrophy 34 and cell reprogramming 33, 38. In these studies, the SAM-based CRISPRa systems were delivered by plasmids 22 or viral vectors such as adeno-associated virus (AAV) or lentivirus 33, 38. However, transfection of plasmids into stem cells is notoriously inefficient, while the SAM system requires the expression of dCas9-VP64, sgRNA and the MPH fusion protein, making it difficult to be incorporated into a single viral vector with limited cloning capacity (e.g. 4.7-5.0 kb for AAV; 8 kb for lentivirus), hence the SAM system needs to be delivered by two separate AAV 34 or lentiviruses 33. Although these systems showed promise in gene activation, concurrent delivery of all the SAM components to the same cells by multiple vectors is in theory less efficient than by a single vector.

In contrast to AAV and lentivirus whose packaging capacity is limited, BV has a packaging capacity of at least 38 kb 39 and can transduce a wide variety of primary cells including bone marrow-derived mesenchymal stem cells and ASC at efficiencies higher than 95% 40, 41. Here we incorporated dCas9-VP64, MPH and a sgRNA array targeting 9 positions on 3 neurotrophic genes (*BDNF*, *GDNF* and *NGF*) into a single BV, thus allowing us to co-deliver all CRISPRa components (12.7 kb) into the same cells at the same time (Fig. 1). In conjunction with the Cre/loxP system, the hybrid BV enabled robust, simultaneous overexpression of BDNF, GDNF and NGF for at least 21 days (Figs. 2A-2C). Furthermore, the BV vector was able to transduce ASC sheets effectively 10, 18, thus allowing us to functionalize the ASC sheets to produce these 3 neurotrophic factors. Such prolonged and potentiated activation of 3 neurotrophic factors in ASC sheets substantiated their ability to recruit SC (Fig. 3) and stimulate neuron proliferation and neurite outgrowth (Fig. 4)* in vitro*. Furthermore, implantation of the CRISPRa-engineered ASC sheets to the sciatic nerve injury site improved the *in vivo* functional recovery (Fig. 5), nerve reinnervation (Fig. 6), axon regeneration and remyelination (Fig. 7). Notably, BV transduction of ASC elicits negligible cytotoxicity and the BV-delivered foreign gene(s) exists within the cells in episomal form without discernable integration and degrades with time 42, 43. These data and findings altogether implicated the potentials and safety of BV for CRISPRa delivery and peripheral nerve repair.

Gene therapy for nerve regeneration is often accomplished by delivery of the complementary DNA (cDNA) encoding a certain neurotrophic factor which, after overexpression, can promote the nerve repair 44-46. Indeed, we have previously employed the hybrid Cre/loxP-based BV to deliver and overexpress GDNF in ASC sheets, which were able to promote peripheral nerve repair 18. In comparison with the cDNA delivery method, the CRISPRa delivery approach conferred a lower GDNF expression level at 2 dpt (≈128 ng/ml in previous study 18 vs. 124.2±12.8 pg/ml in this study) and relatively inferior nerve regeneration, probably because the BV-mediated cDNA delivery was highly efficient such that the copy cumber of *GDNF* gene introduced into cells was tremendously higher. In contrast, the CRISPRa approach required the expression dCas9-VP64 and MPH fusion proteins, which orchestrated with sgRNA to activate *GDNF* in an indirect manner, hence leading to a lower expression level. Nonetheless, the GDNF level elicited by the BV-delivered CRISPRa system was still significantly higher than the expression level (<30 pg/ml) mediated by ASC or ASC-derived SC 44-46, thereby giving rise to effective nerve repair. Furthermore, the CRISPRa system is RNA-guided, programmable, and multiplexing. By changing the 20 nt spacer sequence, one may readily change the target genes to activate. By adding additional sgRNA cassettes (cDNA for each sgRNA is ≈156 bp), one may easily increase the number of genes to activate without the need of cloning the entire gene fragments, which may vary in size from several hundred to several thousand base pairs. This feature allows us to activate multiple genes (*BDNF*, *GDNF* and *NGF*) to synergistically promote nerve regeneration.

Notably, we chose to simultaneously activate *BDNF*, *GDNF* and* NGF* due to their roles in promoting nerve regeneration. The targeting positions were selected based on computer-aided prediction while the number of sgRNA was randomly chosen. These parameters are known to affect the magnitude of activation 47 and may need further optimization. Moreover, GDNF binds the Ret/Ret1 receptor; NGF binds tyrosine receptor kinases A (TrkA) receptor while BDNF binds TrkB receptor. These receptors may be expressed differentially in different neuronal cells 5 and influence the outcome of neurotrophic factor stimulation. Conversely, these 3 factors activate different, but partially overlapping signaling pathways. For instance, NGF activates MAPK/ERK, PI3K/Akt, and PLC-γ pathways while GDNF also signals through the PI3K/Akt pathway 5. As such, these 3 factors may not represent the optimal combination to activate. Aside from these 3 factors, other neurotrophic or growth factors such as ciliary neurotrophic factor (CNTF), artemin, persephin, neurturin, neurotrophin-3 (NT-3), -4 (NT-4), vascular endothelial growth factor (VEGF), fibroblast growth factor (FGF), leukemia inhibitory factor (LIF) and insulin-like factor-1 (IGF-1) are also shown to improve nerve repair (for review see 6, 48). The effects of activating these genes along with GDNF, NGF and BDNF on nerve regeneration may be screened in a combinatorial and systematic manner using the CRISPRa system.

## 4. Conclusions

Taken together, we exploited BV for efficient delivery of CRISPRa for multiplexed, prolonged and robust activation of neurotrophic genes BDNF, GDNF and NGF in rat ASC for >21 days. The ASC sheets functionalized by the BV-delivered CRISPRa system were able to stimulate SC migration, neuron proliferation and neurite outgrowth *in vitro* and improve the functional recovery, nerve reinnervation, axon regeneration and remyelination in the sciatic nerve injury model. These data implicate the potentials of BV-delivered CRISPRa system for ASC sheets engineering and peripheral nerve regeneration.

## Supplementary Material

Supplementary figure and table.
