# Supplementary material for: CRISPR-based Activation of Endogenous Neurotrophic Genes in Adipose Stem Cell Sheets to Stimulate Peripheral Nerve Regeneration
Source: Theranostics. 2019 Aug 14;9(21):6099–111. doi: 10.7150/thno.36790 (PMC6735509; doi:10.7150/thno.36790)
Supplement: Supplementary file 1 — Supplementary figure and table. [file thnov09p6099s1.pdf]

Table. S1. Spacer sequences were designed with highest computed efficacy and specificity score by crispr-era.stanford.edu/, the window of –700 to –50 relative to transcription start site (TSS) was selected for construction of sgRNA.

**Target gene**	**Distance to TSS**	**Spacer sequence (5'→3')**
Bdnf	-75	GAATTCTATTAGGCAAACTC
Bdnf	-138	GGGGAATTAGGGATACCCCA
Bdnf	-208	GTGCGTGAGTTCGCTAGGAC
Gdnf	-113	GGAGCAAGAGCACGCAATCC
Gdnf	-225	GAGGCCGGTGTGCGAGGTGT
Gdnf	-466	GACACCACGTCAAACCGCGC
Ngf	-155	GTCACAGCAGGTGCCCGGCT
Ngf	-186	GTCCCAGGACCCCTACAACC
Ngf	-691	GCAGTCCCAAGCCTTAAGAC

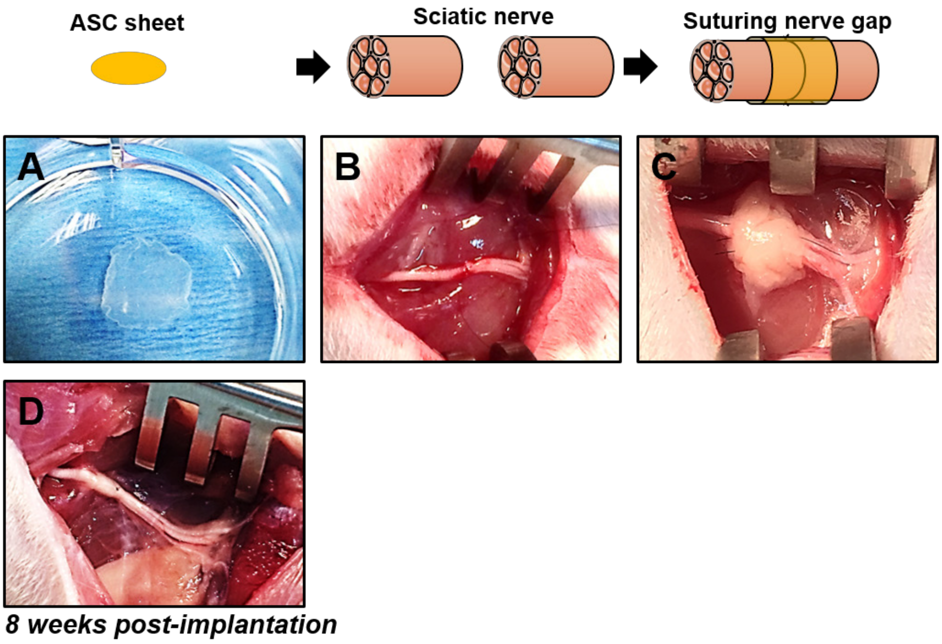

Fig. S1. (A) CRISPRa engineered/mock transduced ASC sheets were prepared and harvested before the animal experiment. (B)The rat sciatic nerve was transected and (C) performed a direct sutured and wrapped with two ASC sheets. (D) The morphology of regenerated sciatic nerve at 8 weeks after implantation.
